# A potentiometric analyser based on the ZX81 microcomputer

**DOI:** 10.1155/S1463924683000449

**Published:** 1983

**Authors:** S. W. Bateson, G. J. Moody, J. D. R. Thomas

**Affiliations:** Applied Chemistry Department Redwood Building UWIST Cardiff CF1 3XA UK


Journal of Automatic Chemistry, Volume 5, Number 4 (October–December 1983), pages 174–181

### A potentiometric analyser based on the ZX81 microcomputer

**S. W. Bateson, G. J. Moody and J. D. R. Thomas**
*Applied Chemistry Department, Redwood Building, UWIST, Cardiff CF1 3XA, UK*

### Introduction

Automation can remove much of the tedium and labour from titration, and the availability of personal computers permits efficient control and the opportunity for fast data-processing at a low cost. In addition, the versatility of the computer allows almost any type of potentiometric determination, including Gran's plot. This paper describes a titration unit controlled by a ZX81 microcomputer through a parallel bus interface system, which can also be used for calibrating electrodes with respect to ion concentration; this is illustrated for a precipitation titration of silver nitrate with bromide using a silver wire indicator electrode and glass reference electrode (Corning Model No. 33 1070 030).

### Instrumentation

The instrumentation layout is shown in figure 1. The titrator employs a Mettler DV11 digital burette, which is driven by a stepper motor, and which, when fitted with a 10 $cm^3$ cylinder assembly (Mettler DV210), can deliver titrant in $mm^3$ ($\mu l$) increments.

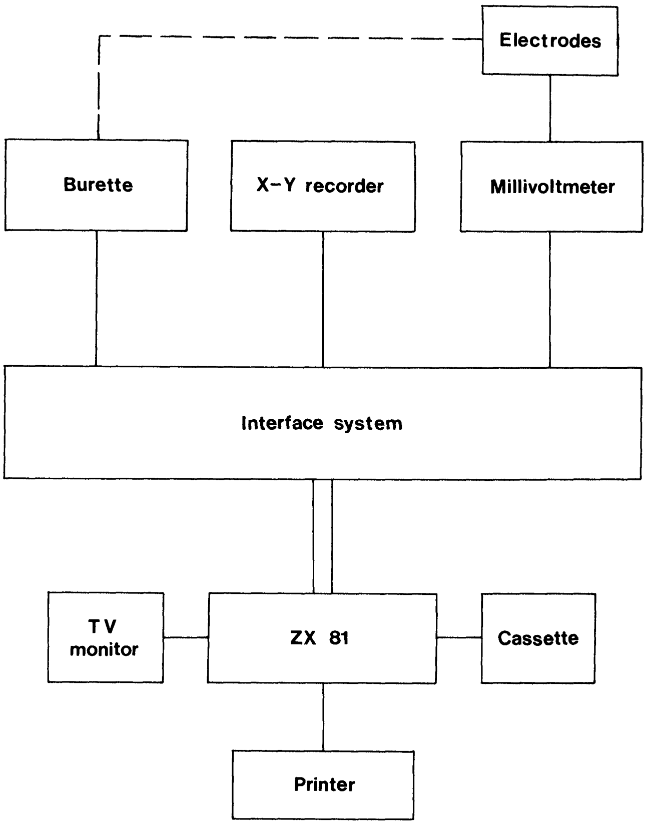

*Figure 1. Auto-titrator system*

The Sinclair ZX81 microcomputer, with television, cassette-recorder and ZX printer, provides the necessary control and processing facilities. Besides being easily obtainable it has the advantages of a powerful Z80 microprocessor, a corrosion-resistant membrane keyboard, and full access to all system lines at the rear-edge connector. It is programmed in BASIC and machine code.

Various potentiometric electrode systems have been used, and their potentials monitored by an Intersil-Datel $4\frac{1}{2}$-digit millivoltmeter (Type 4101L) fitted with a differential high-impedance input buffer.

A Bryans (J-J Instruments type PL3) X-Y recorder is used to make high-resolution plots of titration and derivative curves. With the standard program (obtainable from the authors) three plots (titration curve, second derivative, and calibration curve for direct potentiometry) are drawn on to A3 paper.

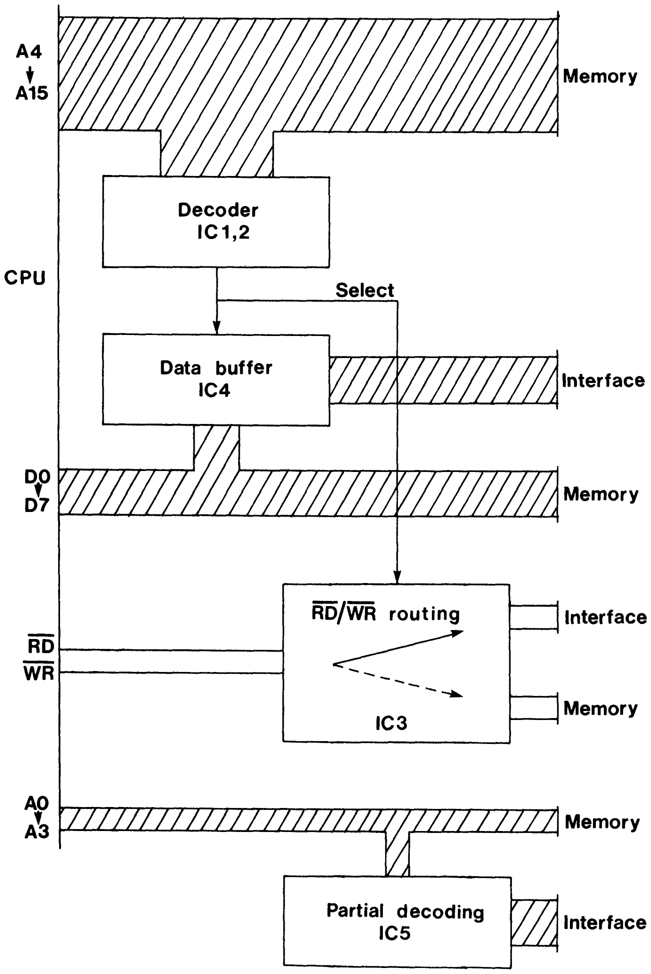

*Figure 2. Address decoder block diagram.*

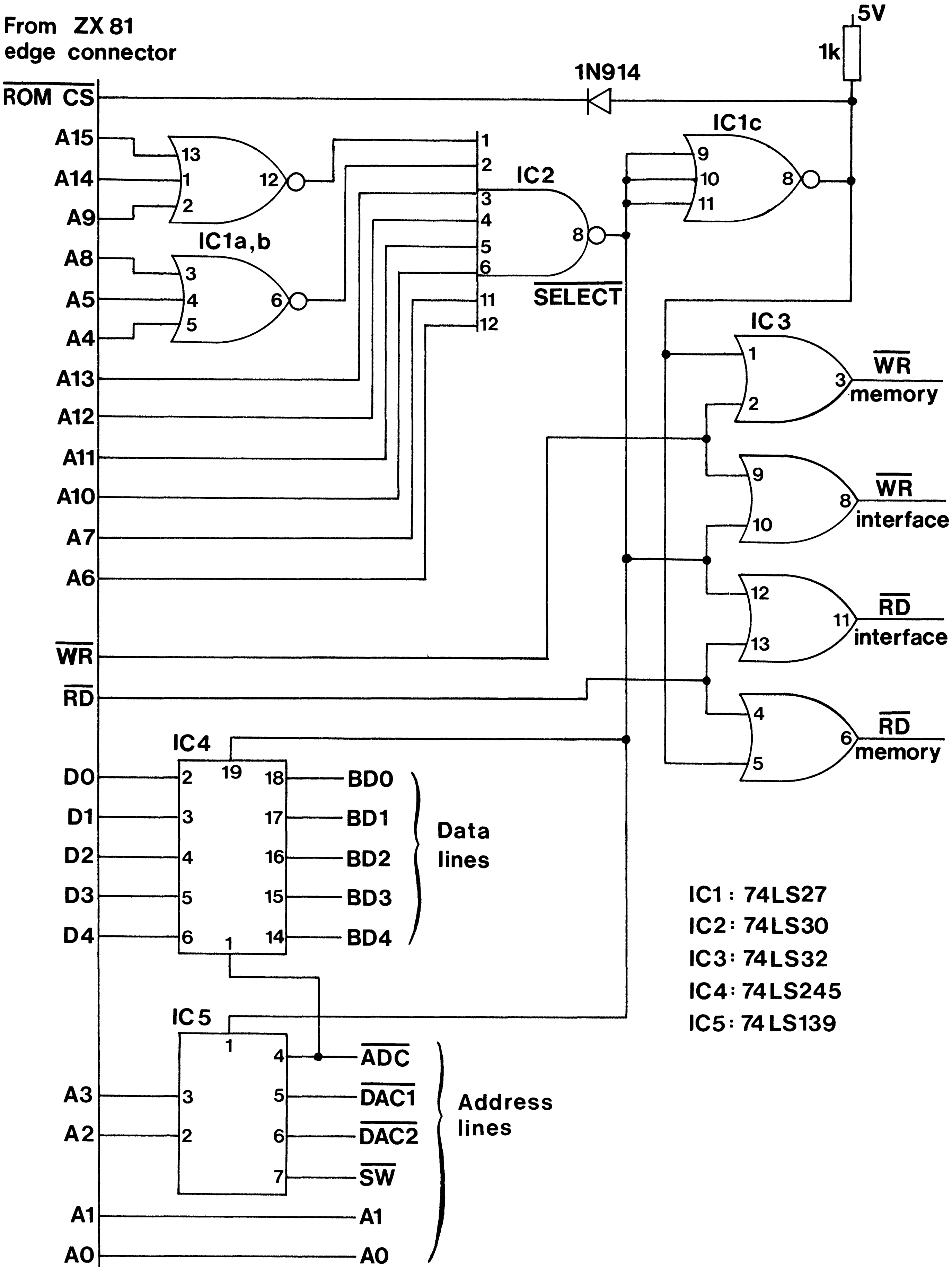

*Figure 3. ZX81 address decoder.*

These units are connected via the interface system (figure 1)—the interface is a parallel bus-oriented system and comparable to the IEEE 488 bus [1]. However, the complexity of the full IEEE standard was considered inappropriate, since this system was conceived as a highly cost-effective addition to the existing data/address bus structure of a Z80-based microcomputer. The design and development of the interface is discussed below.

### *The ZX81 interface system*

The design used was a memory-mapped interface: it is accessed by the CPU in the same way as any normal memory location. The chief advantage of this method is that the circuitry can be operated from BASIC via simple PEEK and POKE commands (PEEK and POKE being BASIC commands to examine or alter, respectively, the contents of specified memory locations). It operates by redefining 16 memory-locations from 15552 to 15567 as interface addresses. The block diagram (figure 2) clarifies this. The circuit diagram is given in figure 3.

When the correct combination appears on the address lines (i.e. A 4, 5, 8, 9, 14, 15 LOW; A 6, 7, 10, 11, 12, 13 HIGH) the decoder (ICI, IC2) activates the 'SELECT' lines (figure 3). These then operate the routing circuit (IC3) and divert the $\overline{RD}$ and $\overline{WR}$ signals away from the memory and into the interface. (Note [*a*] the bars in $\overline{RD}$ and $\overline{WR}$ indicate that these lines go LOW when active, a common feature of control lines, and [*b*] $\overline{RD}$ and $\overline{WR}$ signals always appear shortly after the address is established.) This will occur regardless of the state of the lowest four address lines A0–A3, i.e. for $2^4$ or 16 addresses. In this system, the

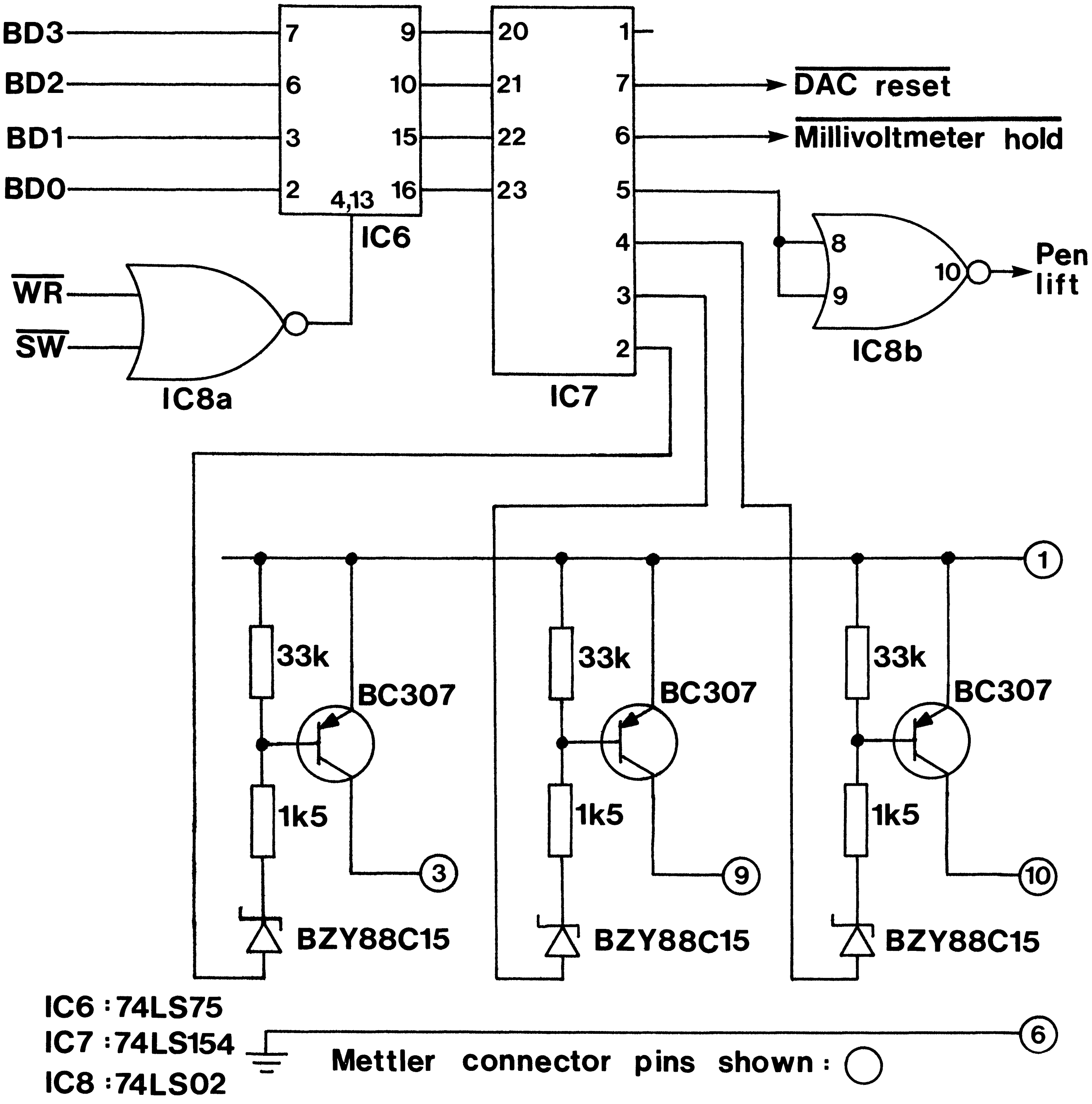

*Figure 4. Digitally controlled switch.*

addresses from 15552 to 15567 are used. These addresses have been shared between four distinct circuit functions, as follows:

15552–15555: analogue-to-digital converter (ADC)
15556–15559: digital-to-analogue converter (DAC1)
15560–15563: digital-to-analogue converter (DAC2)
15564–15567: digitally controlled switch (SW).

These are selected by decoding A2 and A3 into four lines (IC5, figure 3). This leaves A0 and A1 to control the circuit blocks.

*Tri-state logic and buffering*

So that data lines may be shared by all sections of a computer, 'tri-state' logic is used. Here, any part of the circuit which can send a signal to the bus has three possible outputs: low, high, and high-impedance (isolated). Only one device is enabled at a time, thereby avoiding the possibility of two outputs conflicting. Devices are generally enabled under CPU control, via the address and $\overline{RD}$ or $\overline{WR}$ lines etc.

In this design, a bidirectional tri-state buffer (IC4, figure 3) is used to isolate the external circuitry from the ZX81. Since the rest of the interface design consists either of inputs driven by the ZX81 or of tri-state outputs this may seem unnecessary; but since in practice there is a limit to the number of device inputs (and, due to capacitance effects, the length of wire) a Z80 CPU can drive [2], this buffer was found essential. (Most microcomputer systems are fully buffered as standard; the ZX81, for reasons of cost, is not.) The buffer normally isolates in both directions; when the millivoltmeter circuit is addressed it is switched to receive data (by IC5) and when any other part is addressed, it sends data. The remaining circuit blocks are now described in order of complexity.

*Digitally controlled switch*

A single 16-way switch, operated by any of the addresses 15564–15567, has been provided (figure 4).

IC6 (figure 4) is a four-bit latch which acts as a buffer when enabled, but holds its outputs when disabled. It is enabled when

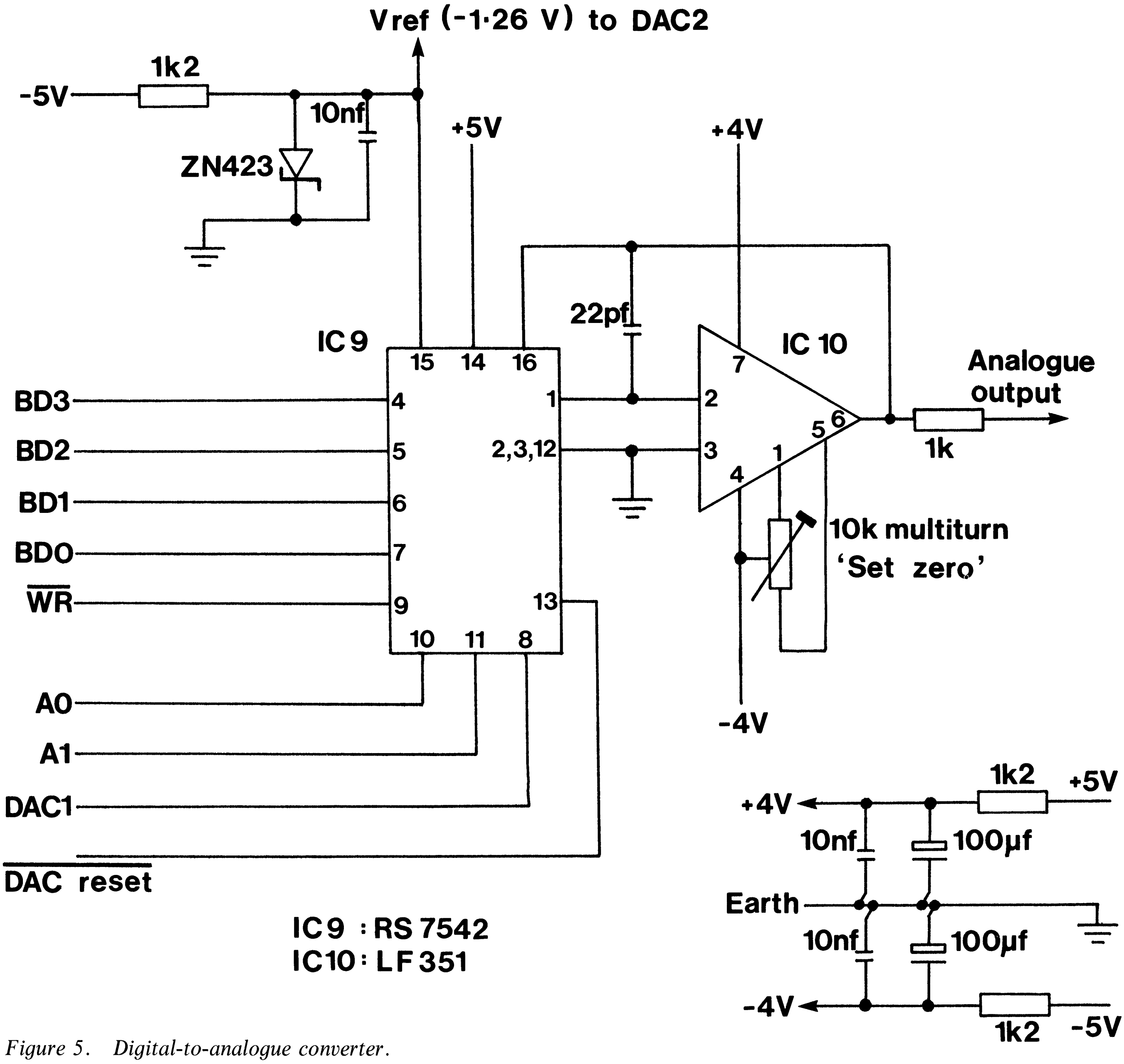

*Figure 5. Digital-to-analogue converter.*

*Table 1. Digital switch functions*

| Function | n | IC7 pin |
|---|---|---|
| Normal (no function) | 0 | 1 |
| Add 0·001 $cm^3$ from burette | 1 | 2 |
| Add 0·1 $cm^3$ from burette | 2 | 3 |
| Refill burette | 3 | 4 |
| Lower chart recorder pen | 4 | 5 |
| Hold millivoltmeter reading | 5 | 6 |
| Reset D-A converters to zero | 6 | 7 |

addressed, i.e. when $\overline{WR}$ and $\overline{SW}$ go low. Data is then passed to IC7, a 4-to-16-line decoder. Addressing the circuit from BASIC by the instruction POKE 15567, n (n = 0–15), causes output n of IC7 to go low, the others remaining high. The seven functions required in this application, together with values for n and output pin numbers for IC7, are given in table 1.

The Mettler burette uses 18 V switching levels, which are driven from 5 V logic by the level-shifting circuits shown on IC7 pins 2, 3 and 4. Pin numbers for the Mettler multi-way connector are also shown.

IC8 (b) is added to invert IC7 pin 5 output since the chart-recorder pen is lowered by a high logic level.

*Digital-to-analogue converters*

The two digital-to-analogue converters (DACs) are identical and share a common voltage reference and power-supply. They are based on the Radiospares Components 12-bit converter, type 7542 (figure 5).

This device is quite easy to use, but because of the eight-bit limitation of the data bus, it must be loaded with data in three sets of four bits each [3]. A fourth operation then combines the bits and generates the analogue output.

Thus, when the first DAC address is selected ($\overline{DAC1}$ low, A0, A1 low) the first converter will expect the four least significant bits to be present on the data bus when the $\overline{WR}$ (Write) line goes low; when the second address is selected (A0 high, rest as before) the device will expect the middle four bits; similarly for the third address and the four most significant bits.

When the fourth address (A0, A1 high) is selected, the data lines are ignored and the data previously collected is transferred to the 12-bit output register of IC9, from which the appropriate analogue voltage is derived.

The $\overline{CLEAR}$ input (figure 5) can be used to reset the 12-bit register to zero, and is operated by the $\overline{SW}$ function described earlier. In the circuit of figure 5, the output voltage ranges from 0 to (−4095/4096) × Vref, so the reference voltage must be negative for a positive output range. The negative supply for the reference and output amplifiers is taken from the analogue-to-digital converter (see below) which has an on-board supply inverter.

*Analogue-to-digital converter (ADC)*

The connection of the Intersil-Datel 4½-digit millivoltmeter to the ZX81 data bus involves dual use of data lines and machine-code programming. This meter gives access to its digital outputs via four data lines and five digit 'strobes' (figure 6).

The display is internally multiplexed [4], i.e. the digits are illuminated sequentially (the most significant first) but with such a high repetition rate (500 Hz) that they appear continuously lit. Thus, at any time, the data lines carry a binary coded number representing just one of the digits, the digit strobe lines indicating which. The computer is required to wait for the first digit line to go high, then read the data lines, store the number, and go back to wait for the next digit, in less than 0·4 ms. This must be done with a short piece of machine code rather than BASIC.

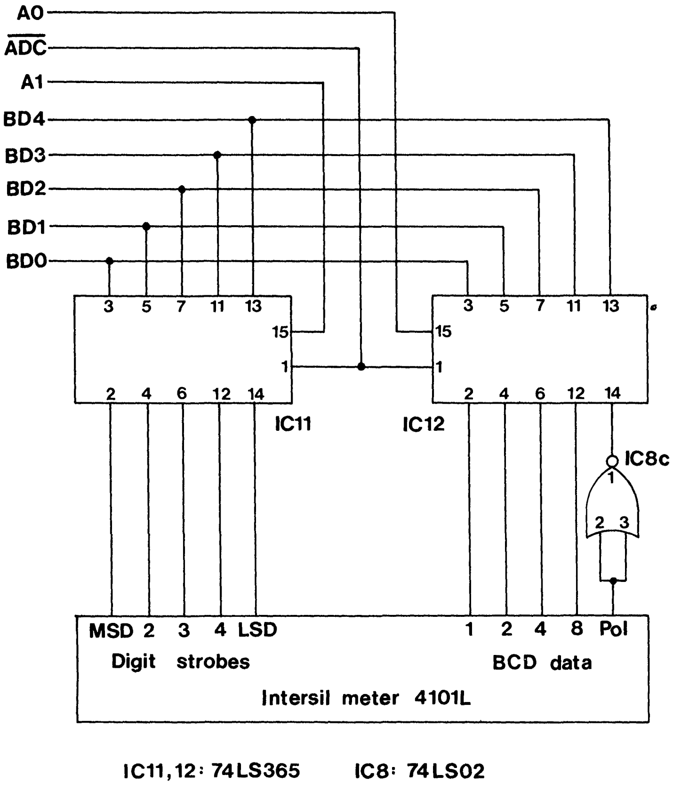

*Figure 6. Analogue-to-digital input adaptor.*

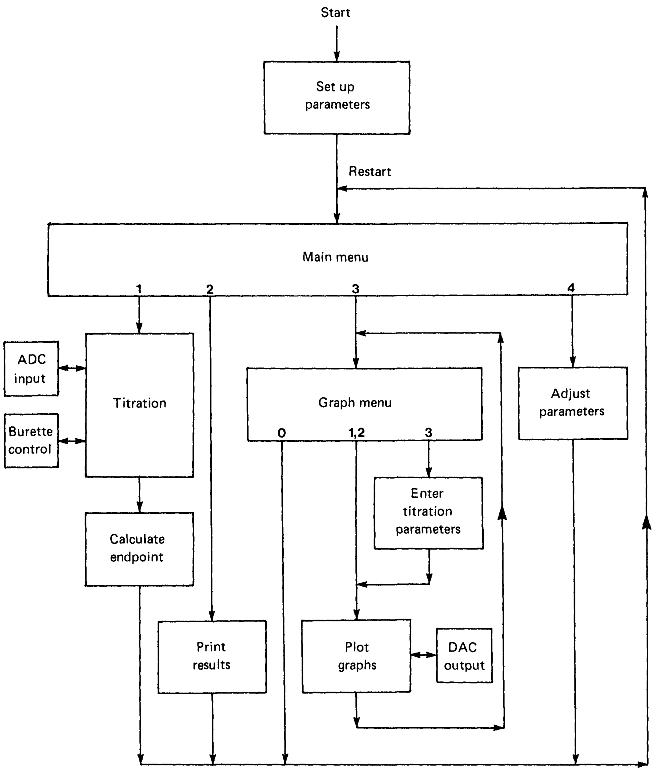

*Figure 7. Program block diagram.*

The data lines are used alternately for examining the digit and data outputs, which are accessed as required through the tri-state buffers IC 11, 12 (figure 6). The required buffer is selected by $\overline{ADC}$, A0 and A1 as shown. The program address of each buffer is chosen so that when selected, its address line will go *low*, while the address line of the other buffer remains high. The polarity signal from the meter, which goes low when negative, is inverted and fed in with the data lines.

*ADC input machine-code subroutine*

The Z80 code subroutine [5] reads the five digits in turn, placing them in five reserved memory locations from 16514 to 16518. The most significant digit is 0 or 1 when positive, 16 or 17 when negative. The BASIC program combines the digits, multiplying them by the appropriate power of 10 to recreate.the original meter reading. When a number higher than 16000 is registered, the program subtracts 16000 and makes the result negative.

### Program and illustrative results

The titration program is constructed in sections, based on subroutines for operating the interface system. The structure is shown in the block diagram (figure 7).

On running the program, the user chooses from a 'menu' of available options.

### *Titration options*

The first main option is the titration, which then proceeds without further attention. It calls on the burette control and ADC input subroutines. Various titration parameters are set up in advance but can be changed by the user. When titrating, the system adds a quantity of titrant, waits a pre-set time then starts taking EMF readings.

When these are stable within a pre-set limit, the EMF, together with titrant volume and derivative values, is stored in an array.

As the endpoint approaches, the addition volume is reduced in response to the increasing slope of the titration curve. The volume of each aliquot is decided as follows:

$$\text{New VOL} = \frac{\text{previous VOL} \times \text{target emf change}}{\text{previous emf change}}.$$

When the titration is complete, the endpoints are calculated from the second derivative values. The first and second derivatives are 'averages' over several titration points to reduce the effect of random errors. Maximum and minimum values are recorded for automatic scaling of graphs. The main menu is then re-presented.

### *Results options*

The second menu option is a display and print-out of titration results, while titration curve plotting is the third option. Typical results for a precipitation titration of silver nitrate with potassium bromide are shown in figure 8.

Besides titration and derivative curves, electrode calibration curves may be drawn, based on the following principles:

(1) At the endpoint of a titration, there is a known small concentration of detectable species in solution, dependent on the solubility product for an insoluble product or the formation constant for a complex.

(2) Knowing the stoichiometry of the reaction, the titrand concentration can be found at all stages of the titration (figure 9).

For instance, at Va (figure 9), the amount of titrand in solution equals (Vep − Va) times the titrant concentration; so the *titrand* concentration can be found by allowing for solvent volume, dilution by titrant, solubility product, and stoichiometry.

Knowing the titrand concentration at each point, plotting log (conc) versus electrode EMF will result in a conventional calibration curve. This is illustrated in figure 10 for a silver ion calibration of a silver wire electrode used in conjunction with a glass electrode as reference. This is generally applicable to various potentiometric indicating electrodes, including ion-selective electrodes, and is far less tedious that the conventional static procedure based on serially diluted standard solutions.

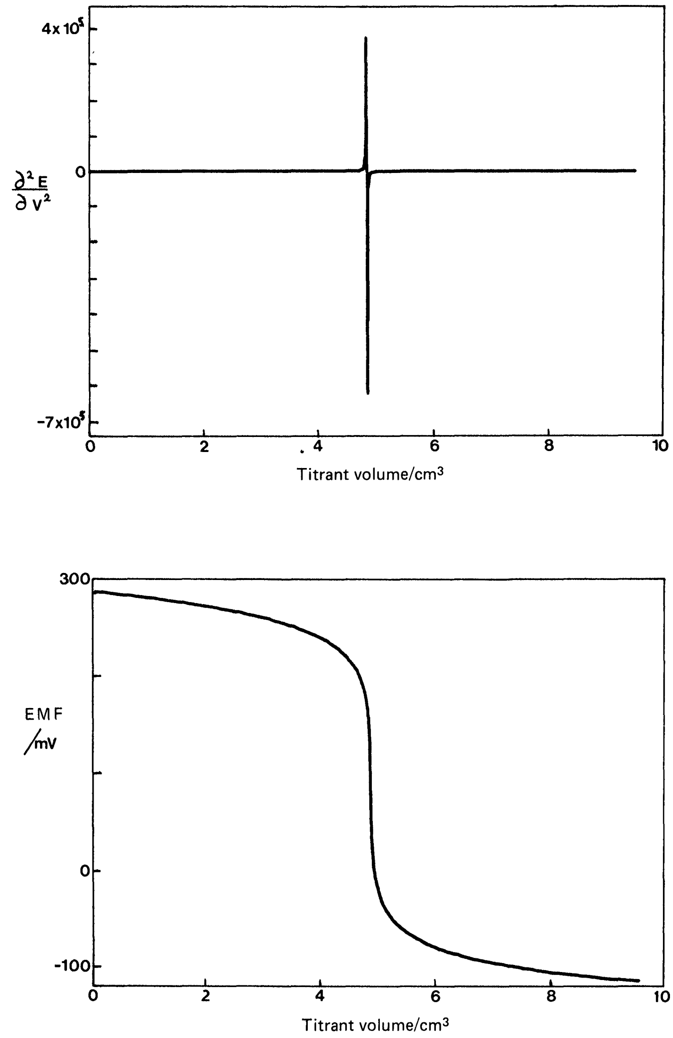

*Figure 8. Titration of silver nitrate (0·5 cm³ of $10^{-2}$ M solution + 20 cm³ deionized water) with potassium bromide ($10^{-2}$ M) using a silver wire indicating electrode with a Corning model 33 1070 030 glass pH electrode as reference. The titration vessel was maintained at 25°C and shielded from light.*

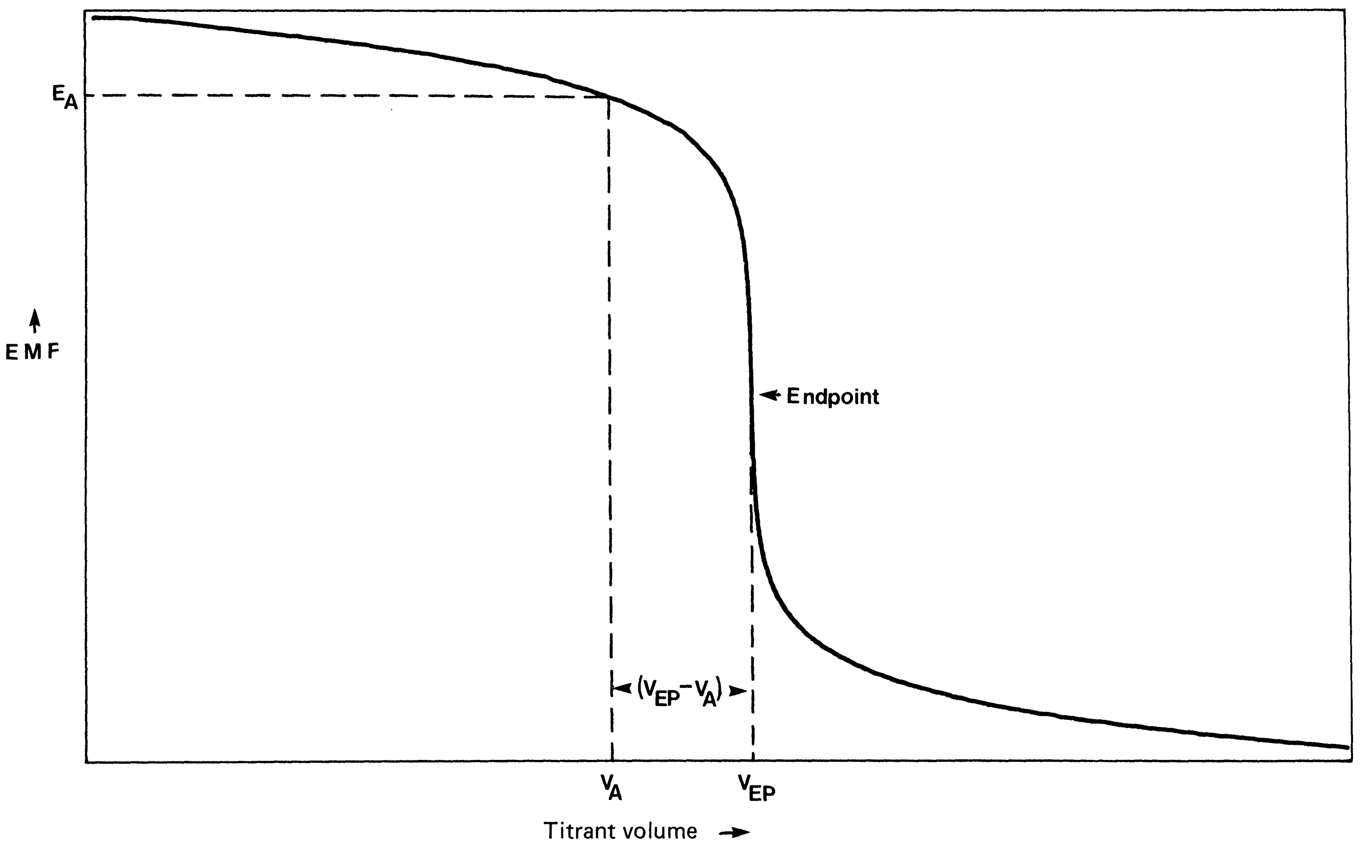

*Figure 9. Calibration calculations from titration curve.*

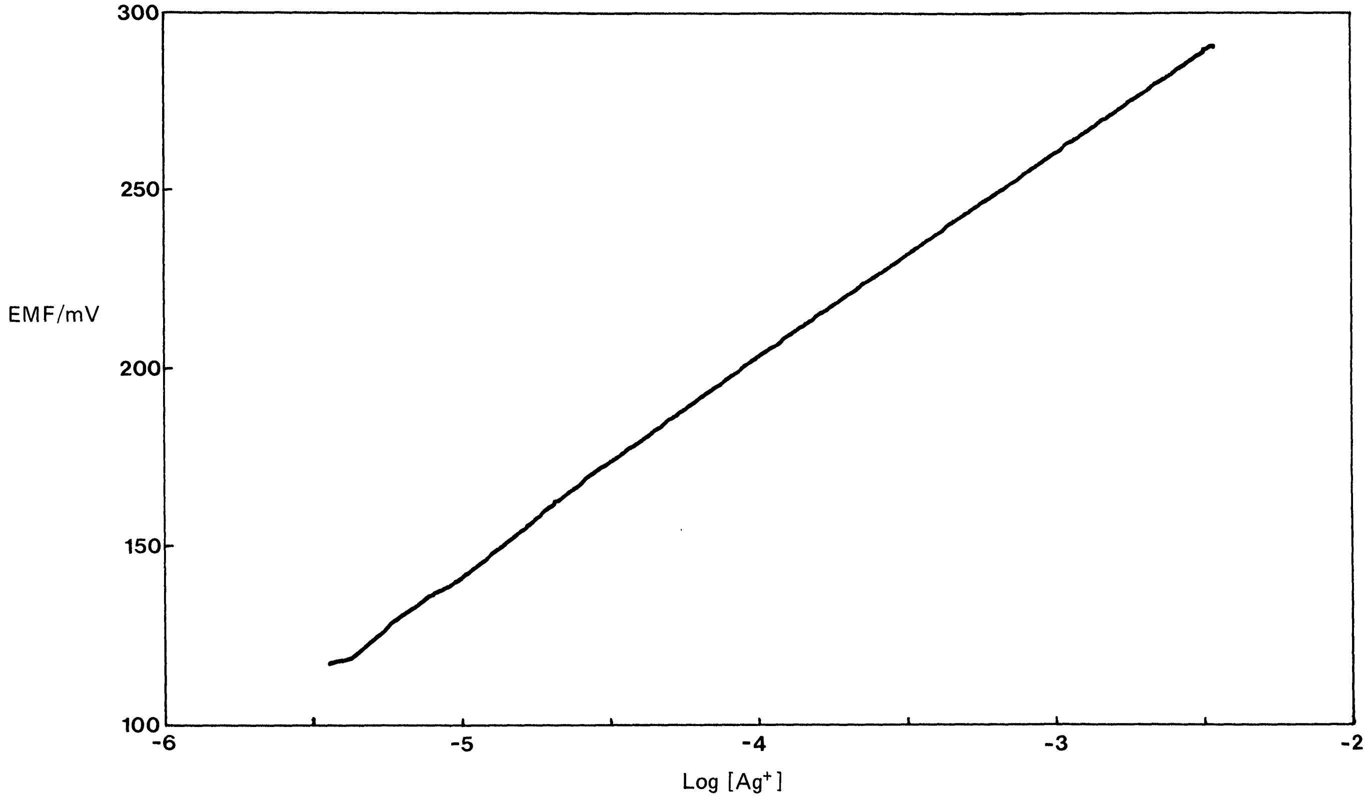

*Figure 10. Calibration of silver wire with respect to silver ion concentration from titration curve of figure 8.*

### Conclusions

The ZX81 is a versatile and economically priced microcomputer which can be conveniently interfaced to a high-precision potentiometric titration assembly. This can be programmed for a variety of options including the facility of setting up electrode calibrations for direct potentiometry.

### Acknowledgements

The authors wish to thank the Science and Engineering Research Council for a studentship (to S.W.B.) under the CASE scheme in conjunction with the Esso Petroleum Company Ltd, Abingdon, UK and especially Mr D. Murtagh for his encouragement. Andrew Woodward and Stuart Davies are also thanked for very helpful discussions.

### References

1. *IEEE Standard 488-1975* (IEEE Inc., 345 East 47th Street, New York, USA).
2. *Z80 Data Book* (SGS-Ates, 1980).
3. *Radio Spares Components Data Sheet R/4399* November 1981).
4. Davies, T. W., *Experimentation with Microprocessor Applications* (Reston, 1980).
5. Zaks, R. *Programming the Z80* (Sybex, 1980).

### UK CHEMISTRY

New titles which might be of interest to readers include:

**Chapman & Hall**
*Practical Absorption Spectrometry: Techniques in Visible and Ultraviolet Spectrometry, Volume 3.* By C. Burgess and A. Knowles (December, *c.* £15·00).

*The Determination of Ionization Constants, A Laboratory Manual.* By A. Albert and E. P. Sergeant (3rd edn, December, *c.* £15·00).

**Longman**
*Analytical Biochemistry.* By David J. Holme and Hazel Peck (October, £25·00).

**Macmillan Press**
'*AnalaR*' *Standards for Laboratory Chemicals.* By AnalaR (7th edn, November, £25·00).

**Scottish Academic Press**
*An Eight Peak Index of Mass Spectra of Compounds of Forensic Interest.* Published for the Forensic Science Society (August, £50·00).

(From Autumn Export Number, *The Bookseller.*)

### ANNUAL CHEMICAL CONGRESS

*Royal Society of Chemistry:*
*16–19 April 1984*

The fourth Society Congress will be held at the University of Exeter, UK; it is to be formally opened by the University's Chancellor, Sir Rex Richards.

Named plenary lectures will be given by: Edward Frankland Lecture—Professor Sir Geoffrey Wilkinson; Robert Robinson Lecture—Professor D. Arigoni; Theophilus Redwood Lecture—Dr R. L. Williams; and Meldola Medal Lecture—Dr A. G. Orpen. The annual conference of Section Secretaries will also be held during the Congress.

The following topics will form the basis of the Congress with the responsibility as indicated:

*Professional—Pro Scientia et Humanitate—The Chemist's Conscience and Duty to Society*
*Analytical—The Analyst in Court*
*Dalton—Dynamic Processes in Inorganic Chemistry*
*Education—Secondary School Chemistry—New Developments and the Future of Chemistry at 16+ and at A-Level (Provisional title)*
Invited lecturers: M. Cox, M. J. Frazer and F. M. Garforth.
*Faraday*—jointly with the *Molecular Solids Discussion Group: Electronic Processes in Thin Films and Novel Conductors*
Conduction; Thin film systems; and Photo-responsive organic systems. Invited lecturers will include: W. J. Albery, D. Bloor, R. H. Friend, H. G. Heller, R. W. Munn, G. G. Roberts, D. R. Rosseinsky, G. Wegner, J. O. Williams, M. R. Willis and J. D. Wright.
*Industrial—Inorganic Chemicals in the Year 2000*
Invited lecturers: S. P. S. Andrew, A. R. Burkin, T. Matthews and B. G. Reuben.
*Perkin—The Science and Art of Synthesis*
*Chemical Information/Historical—Chemical Information: Past, Present and Future*
Invited lecturers: K. P. Barr, W. H. Egan, E. G. Watchurst and I. A. Williams.

It is expected that full meeting registration fees for members will be £40·00 (£70·00 for non-members), whilst the daily fee will be £15·00 (£30·00 for non-members). The fee for accompanying persons will be £10·00. A concessionary fee of £8·00 will also be available for students, schoolteachers, retired members, and unemployed members, and there will be a fee of £50·00 for those attending only the workshop. The charge for accommodation for the three-day period of the Congress (dinner, bed and breakfast) will be about £50·00.

*Further information, including application forms, should be available early in 1984 from Dr John F. Gibson, The Royal Society of Chemistry, Burlington House, London W1V 0BN.*

